# Immunostimulatory effects of *Nannochloropsis oculata* supplementation on Barki rams growth performance, antioxidant assay, and immunological status

**DOI:** 10.1186/s12917-022-03417-y

**Published:** 2022-08-15

**Authors:** A. S. El-Hawy, Haidy G. Abdel-Rahman, M. F. El-Bassiony, Abeer Anwar, Marwa A. Hassan, A. A. S. Elnabtiti, Heba M. A. Abdelrazek, Samar Kamel

**Affiliations:** 1 Animal and Poultry Production Division, Desert Research Center, Cairo, Egypt; 2 Department of Clinical Pathology, Faculty of Veterinary Medicine, Suez Canal University, Ismailia, 41522 Egypt; 3 Immunology and Immunopharmacology Unit, Animal Reproduction Research Institute Cairo, Cairo, Egypt; 4 Department of Veterinary Public Health, Faculty of Veterinary Medicine, Suez Canal University, Ismailia, 41522 Egypt; 5 Department of Animal Wealth Development, Faculty of Veterinary Medicine, Suez Canal University, Ismailia, 41522 Egypt; 6 Department of Physiology, Faculty of Veterinary Medicine, Suez Canal University, Ismailia, 41522 Egypt

**Keywords:** *Nannochloropsis oculata*- Barki rams- performance- Immunity- Comet assay

## Abstract

**Background:**

Natural feed supplements are gaining popularity in the animal production sector due to their safety and potential immunostimulatory properties, as well as the ban of some antibiotics and their negative residual effects. This study was carried out for 1 month to investigate the effect of *Nannochloropsis oculata* supplementation on growth performance and cell-mediated immunological status of rams assessed by leukogram assessment, lipid oxidation product malondialdehyde (MDA), total antioxidant capacity (TAC), interleukin assay after lymphocyte transformation test (LTT) including interleukin 6 (IL6), tumor necrosis factor-alpha (TNF-α), interleukin 12 (IL12), and gamma interferon (γ-IF), as well as Comet assay (% of DNA damage, tail length (px), % DNA in tail, tail moment and Olive tail moment).

**Methods:**

Eighteen Barki rams (26.21 ± 0.64 kg) were divided into 3 equal treatment groups (6 sheep/each), G1: animals served as the control group that was fed the basal diet only, while the other treated groups (G2 and G3 (Nan 1.5% and Nan 3%) were fed the basal diet supplemented with 1.5% and 3% *N. oculata* (dry matter basis), respectively.

**Results:**

The obtained results revealed that G3 showed a significant *(P* < 0.05*) *improvement in performance (body weight and body weight gain), the highest significant count (*P* < 0.05) in lymphocytes, and the lowest significant (*P* < 0.05) levels of neutrophils and neutrophils and lymphocytes ratio (N/L) ratio. Meanwhile, both levels of *N. oculata* significantly (*P* < 0.05) decreased MDA and increased TAC than control which seemed to be directly correlated with supplemented dose. There was a significant (*P* < 0.05) enhancement in the lymphocyte transformation assay produced significant (*P* < 0.05) high cytokines (IL6, γ-IF, IL12, and TNF-α) and the lowest significant (*P* <0.05) percent of DNA damage. The conducted principal component analysis estimated the inter-relationship between parameters and revealed that microalgae correlated strongly with cytokine assay and TAC, and negatively with Comet assay parameters; MDA, and neutrophils.

**Conclusions:**

It can be noted that dietary addition of *N. oculata* 3% increased sheep's performance while also producing significant-high cytokines. It also enhanced sheep immunology by considerably enhancing lymphocyte transformation ability. The antioxidant activity of *Nannochloropsis* appears to influence these findings. It was proposed that the Barki rams’ basal diet be supplemented with 3% *N. oculata*.

## Introduction

Farm-animal health is a global concern because of the predicted increase in meat consumption in the next years. The use of veterinary drugs in livestock production is unavoidable because they are required for disease treatment and prevention of the emergence of new diseases, physiological function modification, growth and productivity improvement, and food safety [1]. Additionally, antibiotic usage has expanded significantly in recent years for the treatment of infectious diseases and agricultural production [2]. However, the administration of veterinary medications in high quantities and constantly may result in the deposition of antimicrobial residues in animal muscle and organs. Consumption of these residues in animal products may endanger consumers’ health, including the development of antibiotic-resistant microorganisms, allergies, reproductive disorders, and hypersensitivity reactions [1]. This issue can be reduced by using natural alternatives, such as supplying plant by-products as feed supplements with appropriate concentrations of antibacterial and health-promoting components [3].

Immunostimulant nutrients can be synthesized or acquired naturally, for example, by ingesting microorganisms such as fungus and bacteria, as well as algae, or even microalgae and seaweed [4]. Generally, natural feed additives are becoming more popular in the animal production sector because of the prohibition on the use of some antibiotics, undesirable residual effects, and economic viability. Microalgae are microscopic microorganisms that quickly emerge as feed components and they have piqued the scientific community’s interest as an excellent source of innumerable crucial elements. They exhibit several biological properties such as antimicrobial [5], anti-inflammatory [6], anticancer [7], and immunomodulatory [8]. Many microalgae are already commercially available, and they are important sources of polyunsaturated fatty acids (PUFA), adding these substances to animal diets improves their general health and immunological condition, productivity, and the quality and stability of the associated animal products. They are also a rich source of almost all of the important minerals and vitamins and have high protein content and digestibility [9]. Consequently, incorporating microalgae in ruminant diets had encouraging outcomes [10]. *Nannochloropsis* microalgae are well recognized in aquaculture nutrition and play a significant role in live feed enrichment due to their high amounts of eicosapentaenoic fatty acid [11]. As a result, they can be used to promote ruminal fermentation and feed digestion [12]. Generally, *Nannochloropsis* spp. has traditionally been employed feeding source, supplying omega-3 fatty acids [13], that contain rumen-protected eicosapentaenoic acid (EPA), docosahexaenoic acid (DHA), conjugated linoleic acid (CLA), and all necessary amino acids required for animal feed [10], also it has been identified as a significant supplier of vital amino acids required by feeding animals [10, 14]. Several studies have highlighted the benefits of using microalgae as natural feed supplements for animals due to their high concentrations of natural antioxidant components such as ascorbic acid, -tocopherol, biotin, folic acid, and pantothenic acid [15]. Furthermore, *Nannochloropsis* contains active ingredients with anti-inflammatory properties, through decreasing TNF-α as well as increasing IL-10 expression [16].

*Nannochloropsis oculata* is among the six microalgae recognized in the genus *Nannochloropsis* and was discovered on the Scotland coast [17]. Multiple in-vitro and in-vivo studies reported *N. oculata*'s positive role in palatability [18], easy digestion [19], immunity [20], antioxidant actions [21], anti-inflammatory and anti-cancer [22]. Simultaneously, several studies have claimed the benefits of *N. oculata*, such as lactating Nubian goats' diets supplemented with *N. oculata* at 5 or 10 g/doe daily increased daily milk output and milk fatty acid profile by raising unsaturated fatty acids (UFA) and C20:5n-3 (-linolenic acid) concentrations and lowering saturated fatty acids (SFA) concentrations, furthermore, *N. oculata* improved nutrient digestion and ruminal fermentation [19]. Additionally, MA Hassan, YK Mahmoud, AAS Elnabtiti, AS El-Hawy, MF El-Bassiouny and HMA Abdelrazek [23] investigated the effect of dietary *N. oculata* (4%) on Barki rams and they recorded that 4% dietary *N. oculata* greatly improves the performance, thyroid hormones, serum biochemical, and antioxidant activity. Moreover, I Abd El-Hamid, W Fouda, H Shedeed, S Moustafa, A Elbaz, S Bakr, B Mosa, A Morsy, A Hasan and K Emam [24] concluded that the addition of microalgae *N. oculata* to rabbit diets improves blood components and improves reproductive, productive, and oxidative status in Hi-Plus doe rabbits.

To the best of our knowledge, there is a lack of information regarding the modulation of the immune system in ruminants when microalgae are used as feed additives, therefore, the main objective of our study is to evaluate the role of *Nannochloropsis oculata* (1.5 and 3%) supplementation on ram growth performance and cell-mediated immunological status as evaluated by body weight, leukogram, interleukins assay after lymphocyte transformation test, oxidative stress markers, and Comet assay.

## Methods

### Sheep and experimental setup

The experiment was conducted on 18 healthy Barki rams (*n* = 6/group) that were maintained for one month from March to April 2021, they were approximately 4 months old, and weighed a mean of 26.21 ± 0.64 kg. At the sheepfold of a private farm near the El-Salam canal in Sahl El Teena, East Qantra area, Ismailia, Egypt, the animals were kept in semi-closed pens, 12 m^2^/6 rams (2 m^2^/ram) for each pen with barriers between groups, with water supplied by troughs and shade available for sun protection throughout the trial. Before the trial began, feces samples were submitted for parasitological analysis to assess the animals’ health [25], and the animals were subjected to a clinical examination, according to WR Kelly [26]. The animals were divided into 3 equal treatment groups (6 sheep/each). G1: Rams in the control group were fed a basal diet as shown in Table 1 [27]. G2 and G3 (Nan 1.5% and 3%): Rams were fed a basal diet that included 1.5 and 3% *N. oculata*, respectively. The experimental sheep were provided with unrestricted food and water access.

**Table 1:** Ingredients and chemical composition of experimental basal diet

Ingredient	(%)	Analyzed chemical com-position	(% on DM basis)
**Wheat straw**	35	**Dry matter**	71.23
**wheat bran**	15	**Crude protein**	12.95
**Egyptian clover**	25	**Total digestible nutrient**	65.85
**broken horse beans**	9	**Neutral detergent fiber**	44.87
**yellow maize**	15	**Acid detergent fiber**	30.35
		**Ether extract**	1.48
		**Calcium**	0.69
		**Phosphorus**	0.43

### *Nannochloropsis* microalgae

The Algal Biotechnology Unit, Biological, and Agricultural Research Division, National Research Centre, Dokki, Giza, Egypt, cultivated and retrieved *Nannochloropsis oculata* microalgae (NNO-1 UTEX Culture LB 2164). K Nuño, A Villarruel-López, A Puebla-Pérez, E Romero-Velarde, A Puebla-Mora and F Ascencio [28] approach was used for controlling the microalga concentration and biochemical composition**.** The strain was grown in an f/2 medium at 21 °C, 30 ppm NaCl, pH 8.2, and under 2X75 W fluorescent lamps. On the sixth day, samples were collected and subjected to centrifugation at 3588 g and 20 °C. The collected microalga was subjected to centrifugation at 897 g and 20 °C for 10 min. The recovered biomass was freeze-dried and kept at 20 °C, separately, until it was used. The chemical composition of *N. oculata* was evaluated using gas chromatography mass at the National Research Centre's complex laboratories in Dokki, Giza, Egypt. Chemical composition (g/100 g) of *N. oculata* includes moisture (7.15), crude protein (55.78), ash (12.29), fat (6.61), and total carbohydrates (18.17). Iron (29.35), sodium (1862.70), zinc (1.02), calcium (229), magnesium (173), and potassium (798) were the quantitative elements of the minerals profile (mg/100 g). Amino acid profile quantitative components (mg/g) include: methionine (69.52), cystine (17.30), phenylanlanine (16.24), lysine (15.20), isoleucine (55.95), leucine (65.11), aspartic acid (30.16), glutamic acid (15.07), histidine (13.22), tyrosine (39.21), threonine (39.21), valine (50.36), serine (11.64), glycine (9.98), proline (31.52), alanine (20.24) and arginine (8.56). In addition, the total polyphenol content was (28 mg gallic acid/100 g) and flavonoids were represented mainly by pyrogallol (179.65 µg/g) and catechin (46.00 µg/g). β-carotene (79.07 (µg/g), vitamin D2 (2.74 µg/g), vitamin D3 (0.41 µg/g) and alpha-tocopherol (10.87 µg/g) were detected. The percentage of antioxidant activity (3.61%) was measured using the 2,2-diphenyl-1-picrylhydrazyl-hydrate (DPPH) free radical test [29].

The algal meal was mixed with the concentrate in the feed mill's mixer. Daily, the concentrate intake was determined based on the feed provided and declined.

### Performance parameters

Rams were weighed at the commencement of the trial to determine their initial body weight (IBwt Kg) and then at the end to get their final body weight (FBwt Kg). Bodyweight gain (BwtGKg) was obtained by subtracting the initial from the final weights and average daily gain (ADG g/head/day) was determined. Feed intake (g DM/head) was recorded daily, and the feed conversion ratio (FCR Kg DM/Kg gain) was calculated to assess the rams’ performance [30].

### Blood sampling, leukogram assessment, and antioxidant markers

At the end of the experimental period, 10 mL of blood samples (*n* = 6/group) were obtained from the jugular vein of rams in different groups and placed in tubes containing EDTA and lithium heparin for each sample/ram. Total and differential leukocyte count (TLC and DLC) were performed for whole blood in EDTA tubes. The procedures were carried out according to BF Feldman, JG Zinkl and NC Jain [31]. The neutrophils/ lymphocytes (N/L) ratio was calculated. The plasma MDA component was measured using a colorimetric kit (# ab118970, Abcam, United Kingdom) and the protocols described by P Agostinho, C Duarte and C Oliveira [32]. TAC plasma levels were measured using colorimetric kit (# K274-100, BioVision, USA) according to O Erel [33].

### Lymphocytes culture and transformation test (LTT)

The buffy coat layer was removed from lithium heparinized blood samples and washed with RPMI-1640 media from Sigma-Aldrich in Egypt. The cleaned cells were resuspended in 1 mL of RPMI-1640 media containing 10% fetal calf serum (Sigma-Aldrich, Egypt). The number of viable lymphocytes per mL of RPMI was determined using a Neubauer Hemocytometer and trypan blue stain (Sigma-Aldrich; St. Louis, MO, USA). Each sample was tested three times. 10 Set up for lymphocytes were cultured with phytohemagglutinin (PHA) mitogen at a concentration of 10 µg/mL. The plates were incubated for 72 h at 37 °C in an incubator with a 5 percent CO_2_ tension. Finally, lymphocyte transformation was carried out at 490 nm utilizing methyl thiazolyl tetrazolium reduction techniques [27]. The supernatant of cultured lymphocytes was used to collect plasma.

### Cytokines assay of PHA stimulated lymphocytes

PHA-stimulated lymphocyte supernatants were tested for interleukin 6 (IL6), tumor necrosis factor-alpha (TNF-α), interleukin 12 (IL12), and gamma interferon (γ-IF) estimation. Later cytokines were measured at 450 nm using ELISA kits supplied by Bioassay Technology Laboratory in China. All laboratory tests, included in the established protocol, were performed following the standard procedures in their enclosed pamphlets.

### Comet assay

The procedures of the Comet assay were followed after the separation of lymphocytes from lithium heparinized blood as described by HMA Abdelrazek, MS Yusuf, SA Ismail and RA Elgawish [27]. The protocol begins with a 2-h incubation of lymphocytes (dimethyl sulphoxide (DMSO) at a final concentration of 1% was used to provide negative controls), followed by gel formation with low-melting agarose and an alkaline electrophoresis solution (1 mM disodium salt of ethylene diamine tetraacetic acid and 300 mM sodium hydroxide, pH > 13) was used for equilibrium, followed by electrophoresis. To reduce the appearance of DNA damage artifacts, the entire operation was carried out under low light. After coding the slides, the analysis was carried out at a magnification of 100 using a light microscope (Nikon, China). Each slide had at least 100 cells examined.

### Statistical analyses

The obtained data were statistically evaluated using the SPSS version 22 computer program (Inc., 1989–2013). The results were presented as means ± standard error for each treatment and were submitted to a one-way ANOVA analysis of variance Duncan’s test to determine whether there was a significant difference between the groups at *p* <0.05. To summarize the primary link between variables, factor analysis was performed as principal components analysis (PCA) using the approach provided by C-WL Liu, Kao-Hung & Kuo, Yi-Ming., Liu, C, K Lin and Y Kuo [34].

## Results

### Sheep performance

Regarding the influence of microalga administration on the performance, Rams supplemented with 3% Nan improved their productive performance measures, as evidenced by a significant (*P<* 0.05) increase in FBwt, BwtGKg, and ADG. They also had the best significant (*P<* 0.05) FCR among the treated groups (Table 2).

**Table 2:** Growth performance of sheep fed on basal diet and others supplemented with *Nannochloropsis oculata* (1.5% or 3%)

Parameter	Experimental groups		
	**Control**	**Nan 1.5%**	**Nan 3%**
**IBwt (Kg)**	25.44 ± 0.75	26.24 ± 0.96	26.96 ± 1.53
**FBwt (Kg)**	30.84^b^ ± 0.55	32.14^ab^ ± 0.90	34.01^a^ ± 1.04
**BwtGKg**	5.40^b^ ± 0.38	5.90^b^ ± 0.16	7.05^a^ ± 0.30
**Average daily gain (ADG) (g/ head/day)**	179.99^b^ ± 13.31	196.67^b^ ± 5.47	234.83^a^ ± 10.55
**Total DM intake/head/day**	925^b^ ± 17.04	964^ab^ ± 28.77	1020.18^a^ ± 33.66
**FCR**	5.36^a^ ± 0.36	4.94^ab^ + 0.20	4.45^b^ ± 0.29

Means with different superscript letters within the same row differ significantly (*P < *0.05)

*IBwt* Initial body weight, *FBwt* Final body weight, *BwtG* Body weight gain, *ADG* average daily gain, *DM* Dry matter, *FCR* feed conversion ratio

### Leukogram assessment

Results illustrated in Table 3 revealed a significant (*P<* 0.05) decline in TLC in the group supplemented with Nan 1.5%. Rams with 3% Nan had the highest significant count (*P<* 0.05) in lymphocytes and the lowest significant (*P<* 0.05) levels of neutrophils and N/L ratio among the treated groups. Both groups supplemented with microalga revealed a significantly increased (*P<* 0.05) monocytes count.

**Table 3:** Leukogram in sheep fed on basal diet and others supplemented with *Nannochloropsis oculata* (1.5% or 3%)

Parameter	Experimental groups		
	**Control**	**Nan 1.5%**	**Nan 3%**
**TLC (10**^**3**^**/µL)**	10.60^b^ ± 0.51	7.44^c^ ± 0.18	13.14^a^ ± 0.62
**Neutrophils (10**^**3**^**/µL)**	46.50^a^ ± 1.20	43.30^a^ ± 2.40	34.60^b^ ± 0.80
**Lymphocytes (10**^**3**^**/µL)**	42.30^b^ ± 0.68	43.60^b^ ± 2.96	52.8^a^ ± 0.74
**Monocytes (10**^**3**^**/µL)**	8.50^b^ ± 0.34	9.60^a^ ± 0.34	9.20^a^ ± 0.34
**Eosinophils (10**^**3**^**/µL)**	2.80^a^ ± 0.25	3.60^a^ ± 0.34	3.20^a^ ± 0.25
**N/L ratio**	1.11^a^ ± 0.04	1.08^a^ ± 0.12	0.66^b^ ± 0.02

Means with different superscript letters within the same row differ significantly (*P < *0.05)

### Malondialdehyde (MDA) and total antioxidant capacity (TAC)

Both investigated concentrations of supplemented *N. oculata* (1.5 and 3%) showed a significant (*P* < 0.05) rise and decrease in MDA and TAC, respectively, as compared to the control. Furthermore, % *Nannochloropsis* supplementation resulted in the lowest and highest significant (*P* < 0.05) levels of MDA and TAC among the treatment groups, respectively (Table 4).

**Table 4:** Malondialdehyde (MDA), total antioxidant capacity (TAC) and Cytokines assay in sheep fed on basal diet and others supplemented with *Nannochloropsis oculata* (1.5% or 3%)

Parameter	Experimental groups		
	**Control**	**Nan 1.5%**	**Nan 3%**
**MDA (nmol/L)**	1.92^a^ ± 0.079	1.72 ^b^ ± 0.02	1.17 ^c^ ± 0.02
**TAC (mmol/L)**	1.19 ^c^ ± 0.06	1.74 ^b^ ± 0.03	2.01^a^ ± 0.03
**LTT**	0.91^b^ ± 0.01	1.03^b^ ± 0.07	1.22^a^ ± 0.07
**IL6 (Pg/mL)**	313.41^b^ ± 5.40	321.45^b^ ± 5.97	393.34^a^ ± 6.68
**γ-IF (Pg/mL)**	137.18^b^ ± 5.87	140.26^b^ ± 5.45	193.47^a^ ± 2.46
**IL12 (Pg/mL)**	139.31^b^ ± 3.5	148.59^b^ ± 4.79	188.20^a^ ± 2.30
**TNF-α (Pg/mL)**	102.54^b^ ± 2.64	105.49^b^ ± 3.37	134.65^a^ ± 1.70

Means with different superscript letters within the same row differ significantly (*P < *0.05)

### Lymphocytes culture and transformation test (LTT)

Lymphocyte transformation is shown in Table 4 demonstrated a significant (*P* < 0.05) increase in Nan 3% groups as compared to other groups.

### Cytokines assay of PHA stimulated lymphocytes

The PHA stimulated lymphocyte in Nan 3% group produced significant (*P* < 0.05) high cytokines (IL6, γ-IF, IL12, and TNF-α) than the control (Table 4).

### Single cell gel electrophoresis (Comet assay)

Data in Table 5 indicated that rams supplemented with Nan 3% had the lowest significant (*P* < 0.05) percent of DNA damage, although both groups supplemented with microalga, in comparison to control, revealed a significant decrease in the remaining parameters associated with the Comet assay (Tail length (px), % DNA in Tail and Olive tail moment).

**Table 5:** Effect of *Nannochloropsis oculata* supplementation on Single cell gel electrophoresis (Comet assay)

	**Control**	**1.5% algae**	**3% algae**
% of DNA damage	11.9^**a**^ ± 0.19	11.06^**b**^ ± 0.17	9.03^**c**^ ± 0.15
Tail length(px)	8.56^**a**^ ± 0.25	5.64^**b**^ ± 0.14	5.84^**b**^ ± 0.16
% DNA in Tail	20.05^a^ ± 0.74	13.38^b^ ± 0.26	14.16^b^ ± 1
Tail moment	1.96^a^ ± 0.26	1.32^b^ ± 0.12	1.78^ab^ ± 0.16
Olive tail moment	1.94^a^ ± 0.07	1.58^b^ ± 0.03	1.6^b^ ± 0.09

Means with different superscript letters within the same row differ significantly (*P < *0.05)

### Principal component analysis (PCA)

A component analysis complex linear correlation was performed to identify the variation in the response to microalgae supplementation with different measured parameters. Components are formed by grouping parameters with significant correlations. The variability of the individual correlation of parameters was estimated using principal component analysis (PCA). The parameters yielded four main components, which explained 86.301 percent of the total variances. The associated variable loadings and explained variance are shown in Table 6. PC1 explained 53.474 percent of the total variance, there was a significant positive loading (> 0.75) on microalgae dose, which correlated strongly with cytokines assay parameters and TAC and moderately with TLC; lymphocyte, and BwtGKg. On the other hand, microalgae dose correlated significantly negatively with Comet assay parameters; MDA, FCR and neutrophils.

**Table 6:** Principal Component Analysis for microalgae dose relationship with the different examined parameters

	**Component**			
	**PC1**	**PC2**	**PC3**	**PC4**
**Microalgae Dose**	0.973	-0.099	-0.117	-0.013
**IL6 (Pg/mL)**	0.911	0.053	0.002	0.241
**TAC (mmol/L)**	0.875	-0.229	-0.238	0.005
**TNF-α (Pg/mL)**	0.861	0.380	0.025	0.110
**γ-IF (Pg/mL)**	0.818	0.099	0.342	0.280
**IL12 (Pg/mL)**	0.798	-0.085	0.121	0.361
**BWG (kg)**	0.697	-0.145	0.458	-0.484
**Lymphocytes (10**^**3**^**/µL)**	0.676	0.530	-0.160	-0.345
**TLC (10**^**3**^**/µL)**	0.509	0.432	0.505	0.302
**LTT**	0.469	-0.493	0.341	0.121
**FI**	0.341	0.588	-0.589	-0.045
**Olive tail moment**	-0.455	0.580	0.436	-0.215
**FCR**	-0.506	0.382	-0.587	0.465
**% DNA in Tail**	-0.566	0.626	0.430	0.057
**Tail length(px)**	-0.685	0.226	0.360	0.425
**Neutrophils (10**^**3**^**/µL)**	-0.761	-0.456	0.094	0.373
**MDA (nmol/L)**	-0.878	0.008	0.169	-0.284
**DNA % of damage**	-0.947	-0.093	-0.112	-0.049
**% of Variance**	53.474	13.581	11.418	7.828
**Cumulative %**	53.474	67.055	78.473	86.301

*PC* Principal component. Strong loading values ≥ 0.75, moderate loading values (0.5–0.75) and (0.3–0.5) weak loading values

## Discussion

The consequence of immune-enhancing feed additives has been established for their beneficial health and performance effects that assist the body in confronting microorganisms and overwhelming them [35]. In recent years, natural products such as marine microalgae have been researched for their antibacterial, antioxidant, anticancer, cardioprotective, anti-inflammatory, anti-diabetic, and anti-hypertensive assets [36]. From an environmental standpoint, the use of microalgae can stand to profit ecological sustainability and nature conservation, particularly water and land preservation, because the required cultivation areas must be restricted. Microalgae are a good potential feed resource for functional animal feeds, their supplementation in animal diets research could pave the way for a new solution to boost human and animal health via better nutrition [9]. In this work, rams were employed as the experimental animals since they are a good model for ruminants and are easy to collect blood from [23]. Additionally, I Altomonte, F Salari, R Licitra and M Martini [37] claimed that ruminants are proper models for microalgae feeding, due to their ability to digest cell wall organisms that are frequently not administrated.

The current study revealed that supplementing *N. oculata* at a 3% concentration increased FBwt and BwtGKg of the studied sheep with improved FCR. Furthermore, microalga dosage directly associated with BwtGKg and negatively with FCR as determined by PCA could support this finding. *Nannochloropsis* has been used in nutraceuticals and feed enrichments [11, 38]; as a rich source of a wide variety of essential and non-essential amino acids as noted in the chemical composition. Amino acids supplementation, especially branched-chain amino acids, exerts a unique capability to recruit signal transduction pathways that up-regulate protein biosynthesis, in skeletal muscle, therefore, increasing BwtGKg [39, 40]. However, the usage of dietary amino acid supplements in ruminants is debatable due to ruminal degradation but other studies consider their dietary role in ruminants [41]. Besides, the zinc contents of the *N. oculata* microalga influence the digestibility of crude protein, organic matter, and acid detergent fiber in sheep [42] as well as intestinal barrier integrity [43], which is positively reflected in growth performance. The iron content of the alga could be incriminated in improved general metabolic status [44]. *Nannochloropsis* zinc as well polyphenols contents are responsible for antioxidants promotion to mitigate oxidative stress that upgrades growth and FCR in sheep [42, 45]. Polyphenolic content (28 mg gallic acid/100 g) of supplemented *N. oculata* could indirectly enhance growth performance via chelating metals that have pro-oxidant potential at the intestinal tract with a lessening of lipid peroxide generation that is achieved through their poor intestinal absorption [45]. Additionally, β-carotene in *N. oculata* (79.07 µg/g) and alpha-tocopherol (10.87 µg/g) are plentiful and this explanation could be supported by WM Aboulthana, AM El-Feky, NE-S Ibrahim, RK Sahu and AE-KB El-Sayed [29] that enhance immune response whereas, there is a strong link between enhanced immunological function and increased weight gain [46–48]. Likewise, previous research has shown that integrating microalgae into livestock animal meals [49] and broiler feed [9, 50] can increase growth.

Additionally, the decrease in N/L ratio was suggestive of *Nannochloropsis*’s stress-relieving impact, particularly at the 3 percent treatment.

In terms of oxidant/antioxidant status, our findings revealed that the addition of *Nannochloropsis* significantly abridged MDA levels while promoting TAC levels as demonstrated by PCA, which showed a negative correlation with MDA and a positive correlation with TAC, respectively. This result could be attributed to the antioxidant ingredients of the used *N. oculata* such as polyphenols (28 mg gallic acid/100 g), alpha-tocopherol (10.87 µg/g), flavonoids (179.65 µg/g), zinc (1.02 mg/100 mg), and β-carotene (79.07 (µg/g) and these results are in accordance with M Jalilian and M Moeini [46]; KKA Sanjeewa, IPS Fernando, KW Samarakoon, HHC Lakmal, E-A Kim, O-N Kwon, MG Dilshara, J-B Lee and Y-J Jeon [22]; D Gessner, R Ringseis and K Eder [45]; WM Aboulthana, AM El-Feky, NE-S Ibrahim, RK Sahu and AE-KB El-Sayed [29]. These active ingredients are contributed to promoted antioxidant activity demonstrated by DPPH. The total antioxidant activity helps to judge the activity in the dominion of stopping life-threatening oxidation [51]. This finding is in accordance with those reported by MA Hassan, YK Mahmoud, AAS Elnabtiti, AS El-Hawy, MF El-Bassiouny and HMA Abdelrazek [23] who found that rams supplemented *N. oculata* 4% showed the lowest significant MDA content. It was shown that the microalga *Nannochloropsis gaditana* can aid in reducing oxidative stress by lowering oxidant indicators like MDA and carbonyl proteins while enhancing antioxidant defense [52]**.** These findings back the notion that *Nannochloropsis gaditana* has the ability to boost antioxidant activity and defend tissues from protein and lipid oxidation [52]. This increase in antioxidant enzymes might be attributed to the neutralization of reactive oxygen species; *Nannochloropsis gaditana* has been shown to scavenge free radicals and reduce lipid peroxidation [53]. Furthermore, *N. gaditana* supplementation decreased oxidative load and improved antioxidants in rats [63]. Sulfated polysaccharides in marine algae are identical to glycosaminoglycan in animals, which reveals their reactivity when administered to animals and has also been found in vitro to exhibit optimal antioxidant action, including both radical-scavenging and metal-chelating capabilities [54]. The enhanced TAC in this study suggested a boosted plasma oxidant/antioxidant balance, which affected weight growth and lymphocyte blastogenesis via preserving DNA integrity [55].

The results demonstrated that pro-inflammatory cytokines were considerably raised in sheep fed with 3% *Nannochloropsis* microalga, although not in the other groups. This denoted the positive immune influence of *Nannochloropsis* on PHA-stimulated lymphocytes. The algal active ingredients such as zinc (1.02 mg/100 g) and iron (29.35 mg/100 g), exert an integral part in enzymes and catalyze their function, therefore, exerting antioxidant and immunostimulant effects [43, 56]. Detected vitamin D (vitamin D2 (2.74 µg/g), vitamin D3 (0.41 µg/g)) in *N. occulata* could be incriminated in physiological processes, for example, on cell differentiation or the immune system [57] therefore, increasing blastogenic activity of PHA-stimulated lymphocytes. On the same trend polyphenols and flavonoid contents in *N. occulata* were proven to provoke an inflammatory response in lipopolysaccharide antigen-stimulated sheep through their antioxidant effect [58]. Moreover, alpha-tocopherol (10.87 µg/g) content and β-carotene (79.07 µg/g) of supplemented *N. oculata* were proposed to stimulate antioxidant potential due to their synergistic action that promoted immune response [59]. This was in harmony with the results of H Herath, DAS Elvitigala, G Godahewa, N Umasuthan, I Whang, JK Noh and J Lee [60], who found that *Nannochloropsis* dietary supplementation levels generated significantly greater overexpression of hepatic IL-1, IL-8, TNF-α, and -IF genes.

Cytokines are crucial in the induction of inflammatory reactions in response to bacterial and viral infections. The innate immune system distinguishes pathogens via toll-like receptors (TLRs) and produces pro-inflammatory cytokines such as TNF-α and interleukins (IL-8 and IL-6). TNF-α is a cytokine that affects pathophysiological processes and it has a role in systemic inflammation by being incorporated into the acute phase response [61]. The current investigators showed that *Nannochloropsis* at a concentration of 3% substantially raised TNF- α following PHA stimulation. IL-12 is a crucial cytokine for activating T-helper 1 (Th1) immune responses by driving NK and T cells to manufacture and release **γ-**IF. The latter is necessary for successful protection against the intracellular pathogens [62]. By activating the hypothalamic–pituitary–adrenal axis, IL-6 stimulates interaction between the neuroendocrine and immunological systems [63]. Furthermore, it oversees intracellular signaling pathway via TLRs that triggers inflammatory cytokines production. In this regard, 3% dietary *Nannochloropsis* shown to have a cell-mediated immunity-promoting impact in PHA-stimulated lymphocytes, which might improve rams' health status toward production. These findings reinforced the PCA-derived substantial positive loading between microalgae dosage and cytokines and BwtG.

Lymphocyte transformation activities are the critical sign of blastogenic activity throughout the development of adaptive immune responses to an infectious agent. Furthermore, this action allows for the continued production of antigen-specific cells during the whole course of the infection [64]. The rate of lymphocytes transformation in sheep treated with 3% *Nannochloropsis* was higher than in control sheep. These findings support *Nannochloropsis*’ immunomodulatory influence on cell-mediated immune response, in which lymphocytes perform a significant role. The effect of β-carotene (79.07 (µg/g) existed in the used *N. oculata,* resulting in increased lymphocyte transformation as well as raised levels of IL-2 and **γ-**IF [65]. Lymphocytes release a variety of cytokines [66], which provide host defense with the capability to counteract many viral illnesses, as seen by higher cytokine levels in the current research. These could lead us to the advantageous possessions of microalga on delayed f hypersensitivity (DH) that were noticed by A Tilwari, N Shukla and PU Devi [67] and A Tilwari, N Shukla and P Devi [68]. DH requires specific identification of the antigen by activated T-lymphocytes, which activate and create cytokines. As a result, capillary permeability, macrophage aggregation and activation, phagocytic activity, and lytic enzyme concentrations increased, resulting in beneficial efficient death [68]. The blastogenic activity of *Nannochloropsis* corresponded with the lower DNA damage percentage seen in the Comet assay as well as declined Tail length (px), and % DNA in Tail and Olive tail moment. Whereas PCA revealed an inverse association between microalgae dosage and Comet assay. In this sense, the oxidant/antioxidant state may play an important impact. Reduced oxidative load caused by alga active ingredients, on the other hand, can increase lymphocyte integrity and competence for cytokine production while decreasing apoptosis, as indicated by reduced Tail length (px), % DNA in Tail and Olive tail moment hence minimizing different innate and cell-mediated caused pathogenic interventions [69].

More researches are needed to determine the best microalgae for specific feeding technology applications, such as supplements in animal diets. Adding these compounds to animal diets improves their overall health and immune status, productivity, as well as the quality and stability of the animal products [9].

## Conclusion

Focusing on the advantageous influences of *N. oculata* dietary inclusion on growth performance and immunological status in sheep, it can be remarked that dietary inclusion with *N. oculata* 3% improved sheep's FBwt and BwtGKg and immunological status in sheep that seem to be mediated via the antioxidant ingredients of *N. oculata that* significantly promoted lymphocyte transformation capacity, DNA integrity and cytokines (IL6, γ-IF, IL12, and TNF-α) production competence against PHA-stimulation.

## Data Availability

All data generated or analyzed during this study are included in this published article [and its supplementary information files].
